# Supplementary material for: Design and Synthesis of Aminopyrimidinyl Pyrazole Analogs as PLK1 Inhibitors Using Hybrid 3D-QSAR and Molecular Docking
Source: Pharmaceuticals (Basel). 2022 Sep 21;15(10):1170. doi: 10.3390/ph15101170 (PMC9610081; doi:10.3390/ph15101170)

Article

Design and Synthesis of Aminopyrimidinyl Pyrazole Analogs as PLK1 Inhibitors Using Hybrid 3D-QSAR and Molecular Docking

Swapnil P. Bhujbal [1,2], Hyejin Kim [1,2], Hyunah Bae [1,2] and Jung-Mi Hah [1,2]*

1 College of Pharmacy, Hanyang University, Ansan 426-791, Korea
2 Institute of Pharmaceutical Science and Technology, Hanyang University, Ansan 426-791, Korea
* Correspondence: jhah@hanyang.ac.kr; Tel.: +82-31-400-5803

Supplementary Material

Table S1: The chemical structures of the selected PLK1 inhibitors with their IC_{50} values.

Dataset 1 : Pyrimidine derivatives

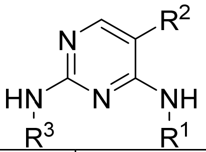

Compound	R¹	R²	R³	IC_{50} (nM)
1			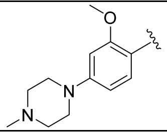	4.67
2*			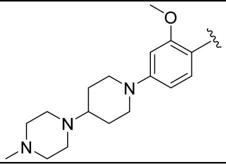	8.13
3*	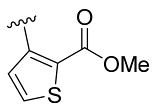	NO_2	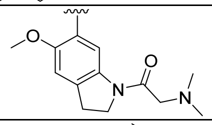	6.19
4			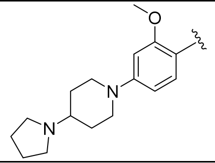	1.58
5			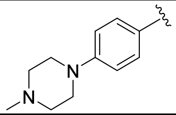	4.37
6*			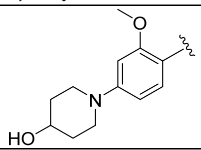	1.27

7			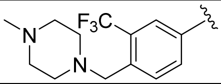	4175
8			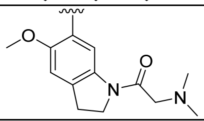	5.13
9			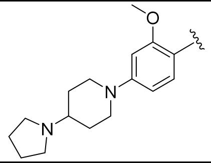	5.26
10*	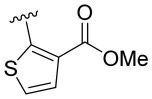		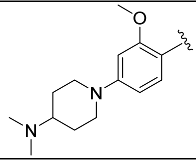	7.35
11			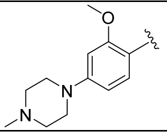	3.69
12			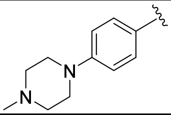	4.99
13			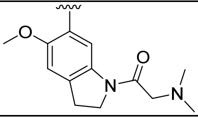	219
14	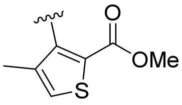		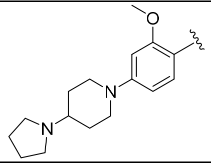	3510
15			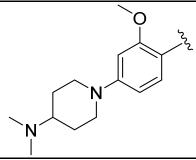	4395
16*	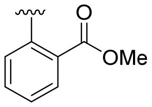		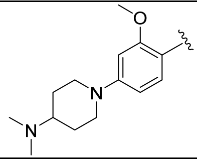	0.577
17			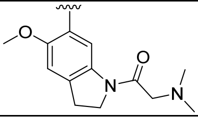	0.508
18*	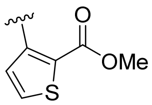	CF_3	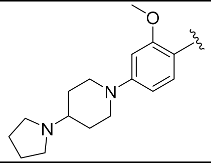	3.31

19			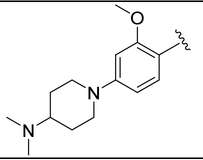	4.55
20			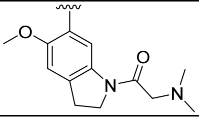	4.87
21*		CN	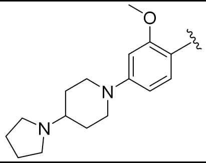	3.82
22			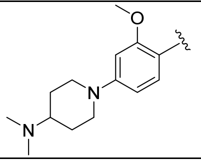	2.67
23			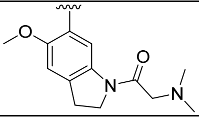	29.3
24*		F	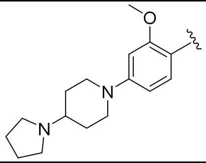	50.2
25*			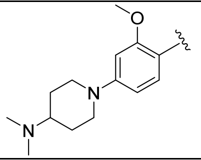	188.5
26			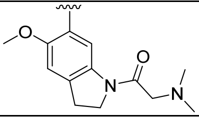	4.28
27		Br	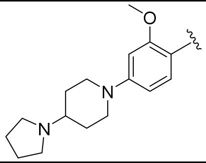	3.44
28			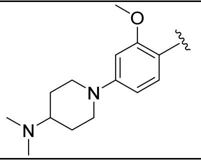	1.39
29		CH_3	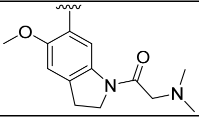	4.72

30*			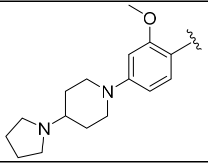	2.22
31			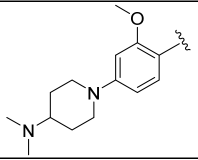	1.85
**Dataset 2 : Quinazoline derivatives**				
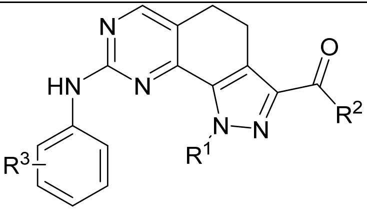 Compounds 32-65				
Compound	R1	R2	R3	IC50 (μM)
32	CH_3	NH2	-	0.068
33	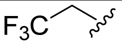		-	0.006
34*	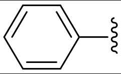		-	10
35	H		-	0.248
36	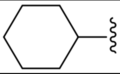		-	0.143
37	*i*-Pr		-	0.430
38	CH_3	OH	-	0.110
39		NHMe	-	4.215
40		NH_2	*o*-CF_3	0.432
41*			*m*-CF_3	0.051
42			*p*-CF_3	0.872
43			*o*-Ac	0.346
44			*m*-Ac	0.100
45			*p*-AC	0.197
46			*o*-OMe	0.042
47			*m*-OMe	0.135
48			*p*-OMe	0.256
49			*o*-NO_2	0.488
50			*o*-Me	0.015
51			*o*-SMe	0.097
52			*o*-NHMe	0.110
53			*o*-F	0.125
54			*o*-*i*-Pr	0.365
55			*o*-CO_2Me	1.117
56*			*o*-CONH2	2.076

57*			*o*-SO2NH2	3.733
58*			*o*-Ph	1.565
59			*o*-OPh	0.278
60			*o*-benzyl	0.943
61			*o*-NHPh	0.949
62			*o*-benzoyl	1.969
63*			*o*-SPh	2.033
64			*o*-NH2	0.150
65			*o*-NHAc	2.523

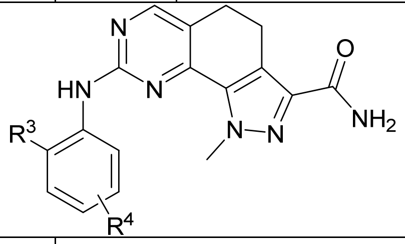

Compound	R^3	R^4	IC_{50} (μM)
66	Ac	3′-(4-methyl-piperazin-1-yl)	2.051
67*		4′-(4-methyl-piperazin-1-yl)	0.464
68*		5′-methyl-piperazin-1-yl	0.109
69	OMe	4′-(4-methyl-piperazin-1-yl)	0.040
70		5′-(4-methyl-piperazin-1-yl)	0.007

* denotes test set compounds in Table S1.

Table S2: CoMSIA models developed using different combinations of fields.

**CoMSIA**	q^2	**ONC**	**SEP**	r^2	**SEE**	**F value**
S	0.543	6	0.821	0.820	0.515	47.777
E	0.414	6	0.929	0.755	0.601	32.290
H	0.521	3	0.821	0.772	0.566	74.597
A	0.357	2	0.944	0.487	0.849	20.910
D	0.367	1	0.930	0.408	0.899	46.901
SE	0.495	6	0.863	0.817	0.519	46.902
EH	0.544	6	0.820	0.850	0.470	59.560
EA	0.405	6	0.937	0.767	0.587	34.493
ED	0.407	6	0.935	0.782	0.567	37.698
SH	0.492	4	0.852	0.793	0.543	62.427

SA	0.459	6	0.893	0.794	0.551	40..529
SD	0.452	6	0.898	0.860	0.454	64.557
HA	0.480	5	0.869	0.848	0.474	58.495
HD	0.492	3	0.845	0.787	0.547	81.452
**SEH**	**0.540**	**6**	**0.824**	**0.855**	**0.462**	**61.993**
SEA	0.470	6	0.884	0.827	0.505	50.133
SED	0.468	6	0.885	0.850	0.471	59.414
EHA	0.534	6	0.828	0.866	0.444	68.068
EHD	0.518	6	0.843	0.879	0.423	75.941
SHA	0.474	5	0.874	0.826	0.503	60.653
SHD	0.465	4	0.884	0.812	0.518	70.353
EAD	0.364	6	0.968	0.772	0.580	35.526
HAD	0.456	5	0.889	0.835	0.490	64.586
SEHD	0.515	6	0.846	0.880	0.421	76.829
SEHA	0.533	6	0.830	0.871	0.436	71.024
SEAD	0.438	6	0.910	0.835	0.493	53.227
EHAD	0.516	6	0.845	0.884	0.413	80.223
SHAD	0.447	5	0.895	0.844	0.475	69.484
SEHAD	0.518	6	0.843	0.889	0.404	84.316

Table S3: The predicted pIC_{50} and residual values for both CoMFA and CoMSIA (SEH) models.

Compound	Actual pIC_{50}	CoMFA		CoMSIA (SEH)	
		Predicted pIC_{50}	Residual	Predicted pIC_{50}	Residual
01	8.331	8.548	-0.217	8.546	-0.215
02	8.090	8.515	-0.425	8.723	-0.633
03	8.208	7.779	0.429	7.702	0.506
04	8.801	8.526	0.275	8.853	-0.052
05	8.360	8.039	0.321	8.002	0.358
06	8.896	8.659	0.237	8.608	0.288
07	5.379	5.340	0.039	5.183	0.196
08	8.290	8.477	-0.187	8.397	-0.107
09	8.279	7.964	0.315	7.828	0.451

10	8.134	7.978	0.156	7.930	0.204
11	8.433	8.797	-0.364	8.714	-0.281
12	8.302	8.299	0.003	8.421	-0.119
13	6.660	6.554	0.106	6.599	0.061
14	5.455	5.386	0.069	5.404	0.051
15	5.357	5.526	-0.169	5.550	-0.193
16	9.239	9.048	0.191	8.906	0.333
17	9.294	8.904	0.390	9.027	0.267
18	8.480	8.799	-0.319	8.469	0.011
19	8.342	8.812	-0.470	8.721	-0.379
20	8.312	8.197	0.115	7.991	0.321
21	8.418	8.614	-0.196	8.149	0.269
22	8.573	8.628	-0.055	8.401	0.172
23	7.533	8.114	-0.581	8.055	-0.522
24	7.299	7.538	-0.239	8.214	-0.915
25	6.725	6.552	0.173	6.466	0.259
26	8.369	8.217	0.152	8.410	-0.041
27	8.463	8.647	-0.184	8.569	-0.106
28	8.857	8.660	0.197	8.820	0.037
29	8.326	8.255	0.071	8.153	0.173
30	8.654	8.695	-0.041	8.312	0.342
31	8.733	8.706	0.027	8.564	0.169
32	7.167	6.543	0.624	6.557	0.610
33	8.222	7.487	0.735	7.888	0.334
34	5.000	5.739	-0.739	5.825	-0.825
35	6.606	6.566	0.040	6.549	0.057
36	6.845	6.936	-0.091	6.742	0.103
37	6.367	6.571	-0.204	6.459	-0.092
38	6.959	6.677	0.282	6.772	0.187
39	5.375	6.281	-0.906	6.626	-1.251
40	6.365	6.583	-0.218	6.522	-0.157
41	7.292	6.032	1.260	7.161	0.131
42	6.059	6.405	-0.346	6.280	-0.221
43	6.461	6.600	-0.139	6.379	0.082
44	7.000	6.916	0.084	6.642	0.358
45	6.706	7.005	-0.299	6.706	0.000
46	7.377	7.007	0.370	6.814	0.563
47	6.870	6.485	0.385	6.685	0.185
48	6.592	6.643	-0.051	6.777	-0.185
49	6.312	6.558	-0.246	6.728	-0.416
50	7.824	6.895	0.929	6.811	1.013
51	7.013	6.840	0.173	6.792	0.221
52	6.959	6.842	0.117	6.705	0.254
53	6.903	6.506	0.397	6.558	0.345
54	6.438	6.853	-0.415	6.861	-0.423

55	5.952	6.399	-0.447	6.125	-0.173
56	5.683	6.626	-0.943	6.023	-0.340
57	5.428	5.915	-0.487	6.467	-1.039
58	5.805	6.784	-0.979	6.684	-0.879
59	6.556	6.334	0.222	6.259	0.297
60	6.025	6.512	-0.487	6.437	-0.412
61	6.023	6.369	-0.346	6.453	-0.430
62	5.706	5.874	-0.168	5.825	-0.119
63	5.692	6.394	-0.702	6.217	-0.525
64	6.824	6.809	0.015	6.708	0.116
65	5.598	5.528	0.070	6.170	-0.572
66	5.688	5.425	0.263	5.686	0.002
67	6.333	6.423	-0.090	7.093	-0.760
68	6.963	7.858	-0.895	8.029	-1.066
69	7.398	7.294	0.104	7.594	-0.196
70	8.155	8.454	-0.299	8.475	-0.320

Table S4: Chemical structures and predicted pIC_{50} activity of designed PLK1 inhibitors.

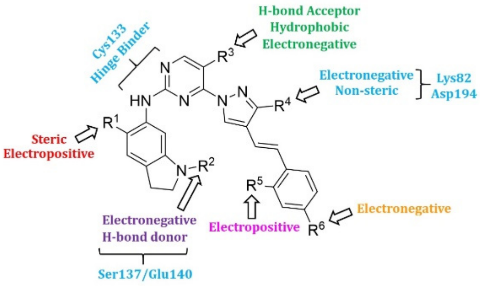

<table>
<tr><th>Compound Structure</th><th>Name</th><th>R¹</th><th>R²</th><th>R³</th><th>R⁴</th><th>R⁵</th><th>R⁶</th><th>Predicted pIC50</th></tr>
<tr><td colspan="9">R¹ Only</td></tr>
<tr><td>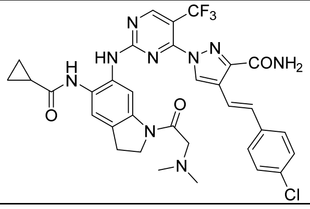</td><td>D1</td><td>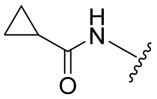</td><td rowspan="2">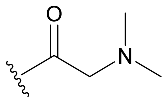</td><td rowspan="2">CF3</td><td rowspan="2">CONH2</td><td rowspan="2">H</td><td rowspan="2">Cl</td><td>9.037</td></tr>
<tr><td>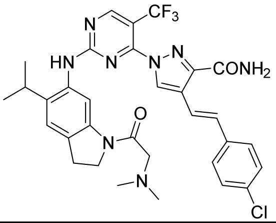</td><td>D2</td><td>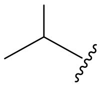</td><td>9.013</td></tr>
<tr><td colspan="9">R² Only</td></tr>
</table>

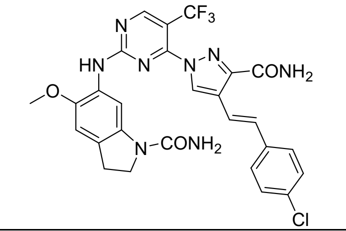	D3	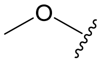	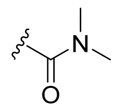	CF_3	$CONH_2$	H	Cl	10.217
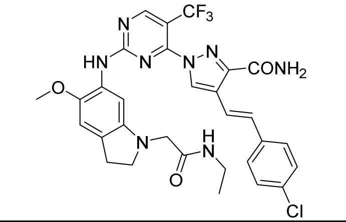	D4		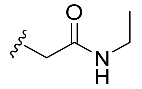					9.309
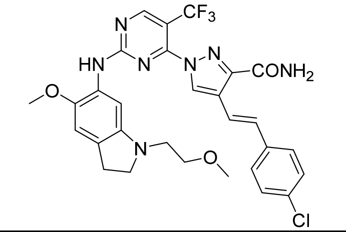	D5		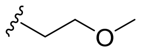					9.715
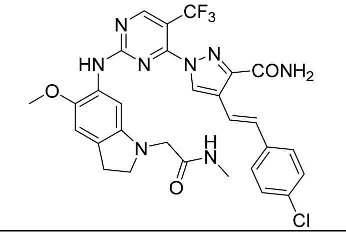	D6		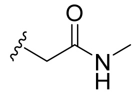					9.223
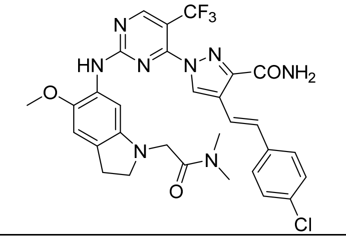	D7		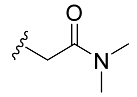					9.394
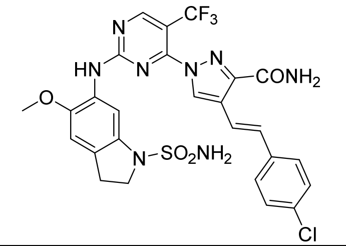	D8		SO_2NH_2					9.418

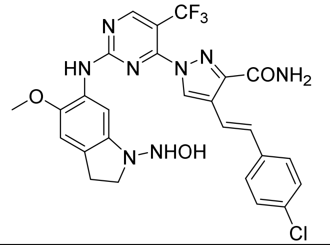	D9		NHOH						9.509
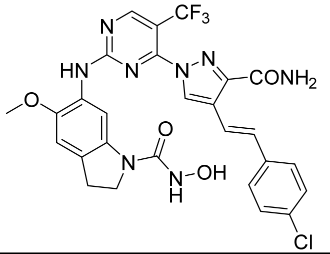	D10		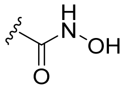						10.272
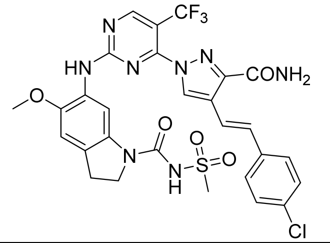	D11		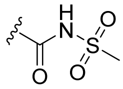						9.829
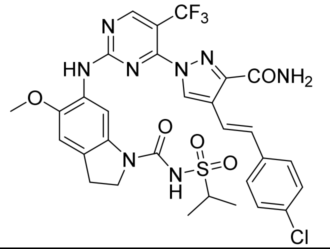	D12		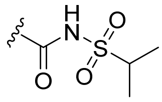						9.914
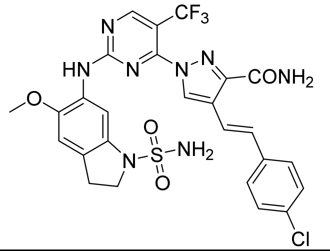	D13		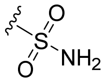						9.422
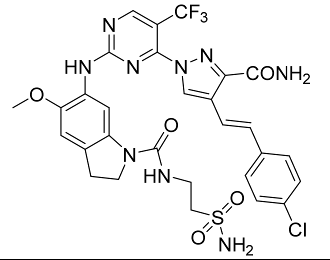	D14		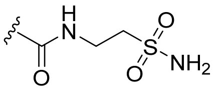						9.811

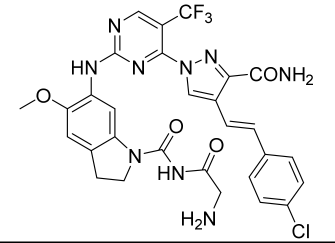	D15		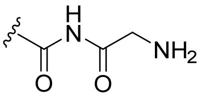					9.709
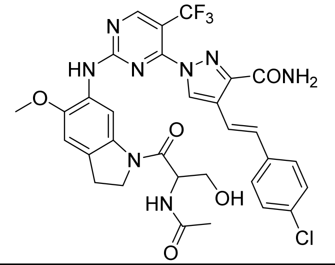	D16		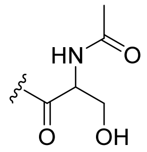					9.66
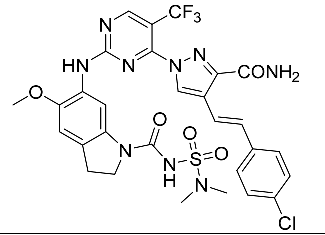	D17		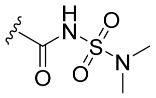					9.903
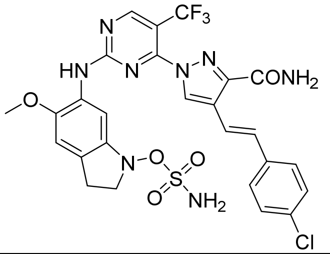	D18		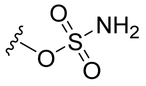					9.269
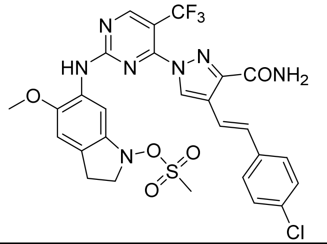	D19		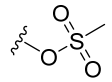					9.317
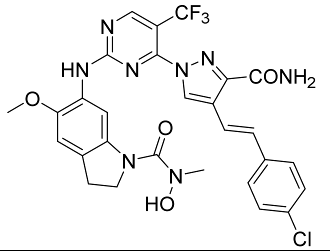	D20		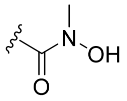					10.056

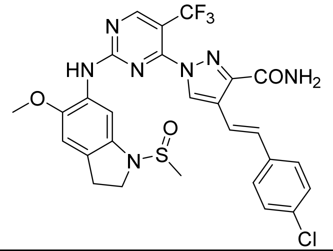	D21		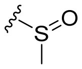					9.428
R[1] + R[2]								
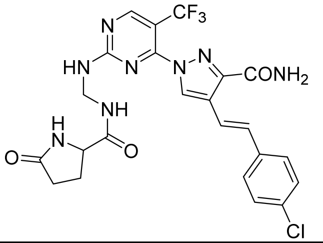	D22	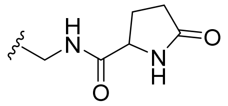		CF3	CONH2	H	Cl	9.221
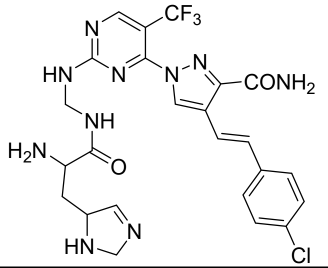	D23	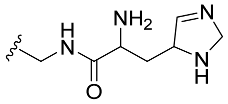						10.188
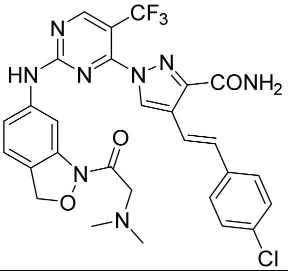	D24	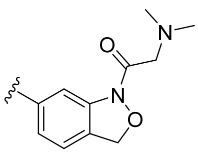						9.544
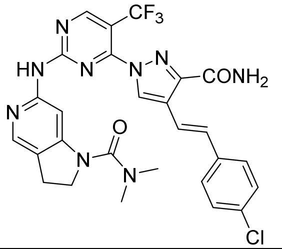	D25	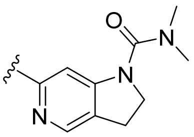						9.411
R[3] Only								
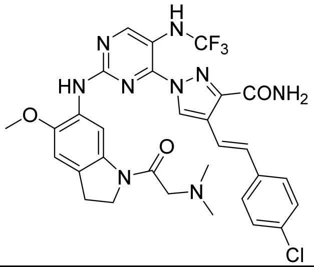	D26	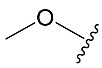	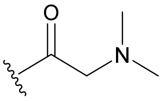	NHCF3	CONH2	H	Cl	9.029

R⁴ Only								
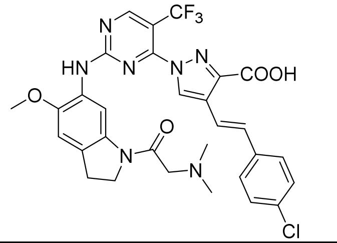	D27	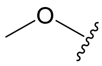	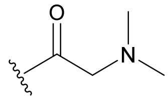	CF_3	COOH	H	Cl	9.052
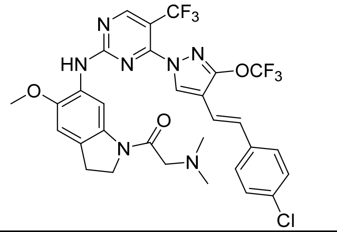	D28				OCF_3			9.133
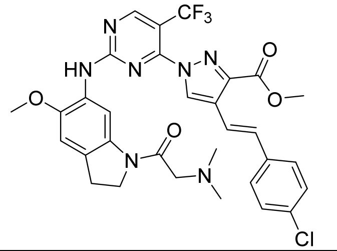	D29				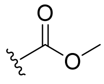			9.067
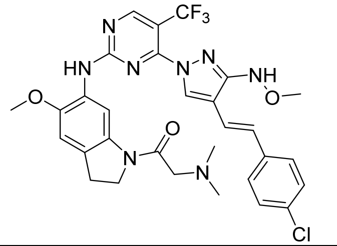	D30				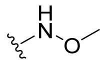			9.114
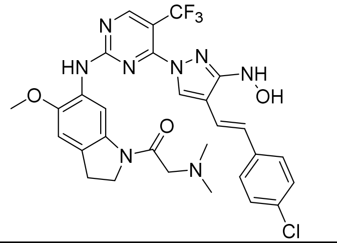	D31				NHOH			9.123
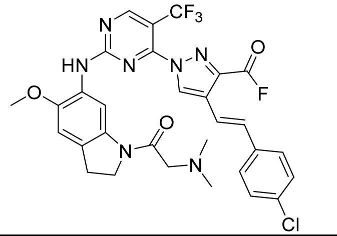	D32				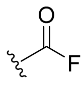			9.081

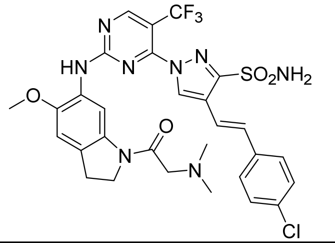	D33				SO_2NH_2			9.059
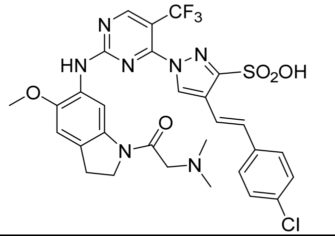	D34				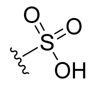			9.059
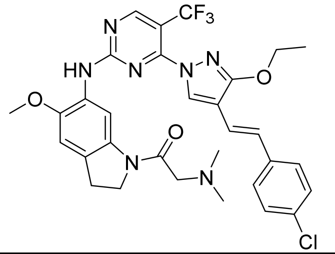	D35				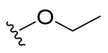			9.163
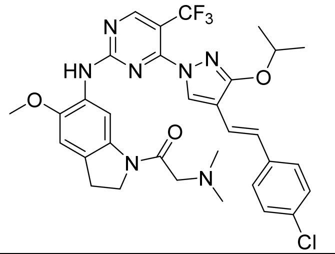	D36				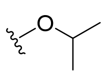			9.151
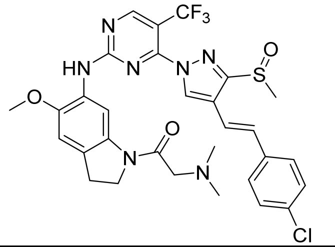	D37				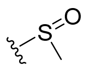			9.118
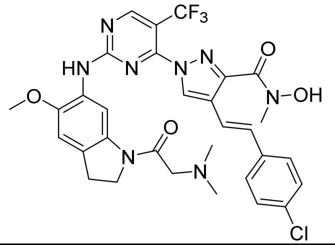	D38				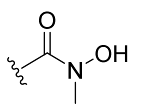			9.047

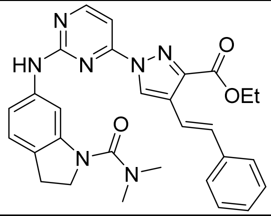	D39	H	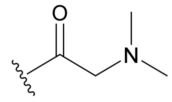	H	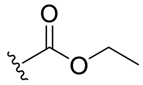	H	H	9.45
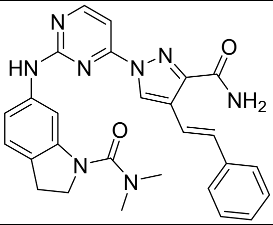	D40	H	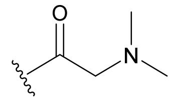	H	$CONH_2$	H	H	9.89

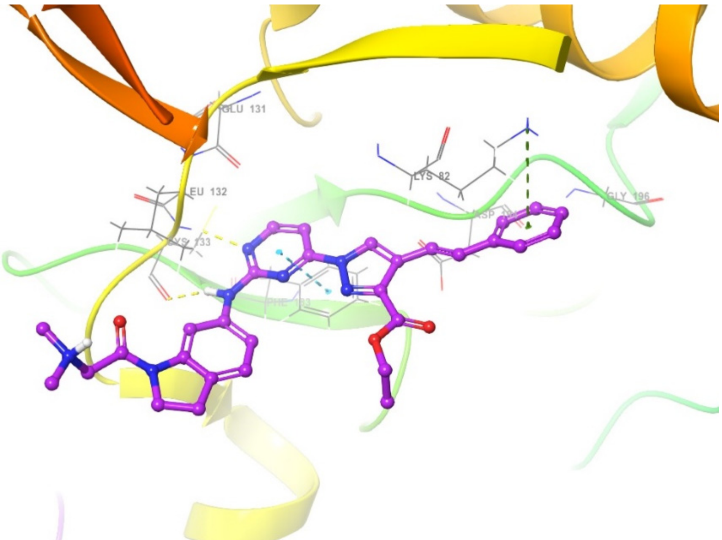

Figure S1. Induced Fit docked pose of the designed compound 39, within the active site of PLK1 (Hydrogen bonds are represented as yellow dotted lines, pi-pi interactions are represented as cyan dotted lines; docking score = -11.284).

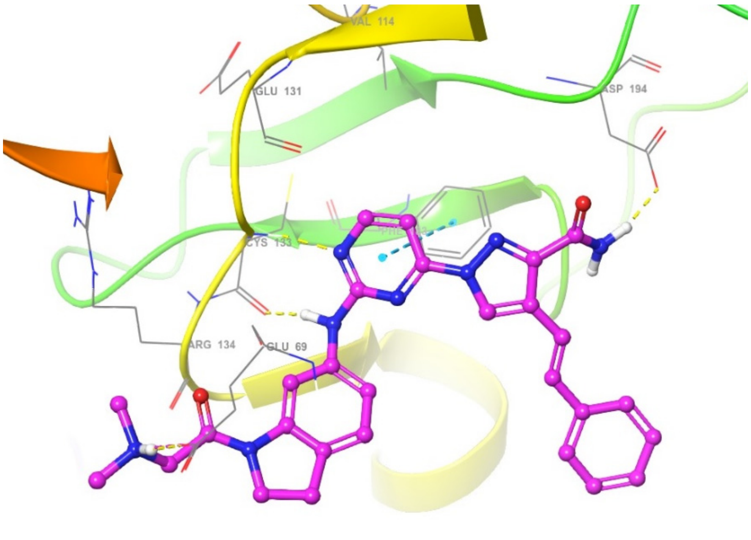

Figure S2. Induced Fit Docked pose of the designed compound 40, within the active site of PLK1 (Hydrogen bonds are represented as yellow dotted lines, pi-pi interactions are represented as cyan dotted lines; docking score = -11.753).

^{1}H NMR Spectra

D39

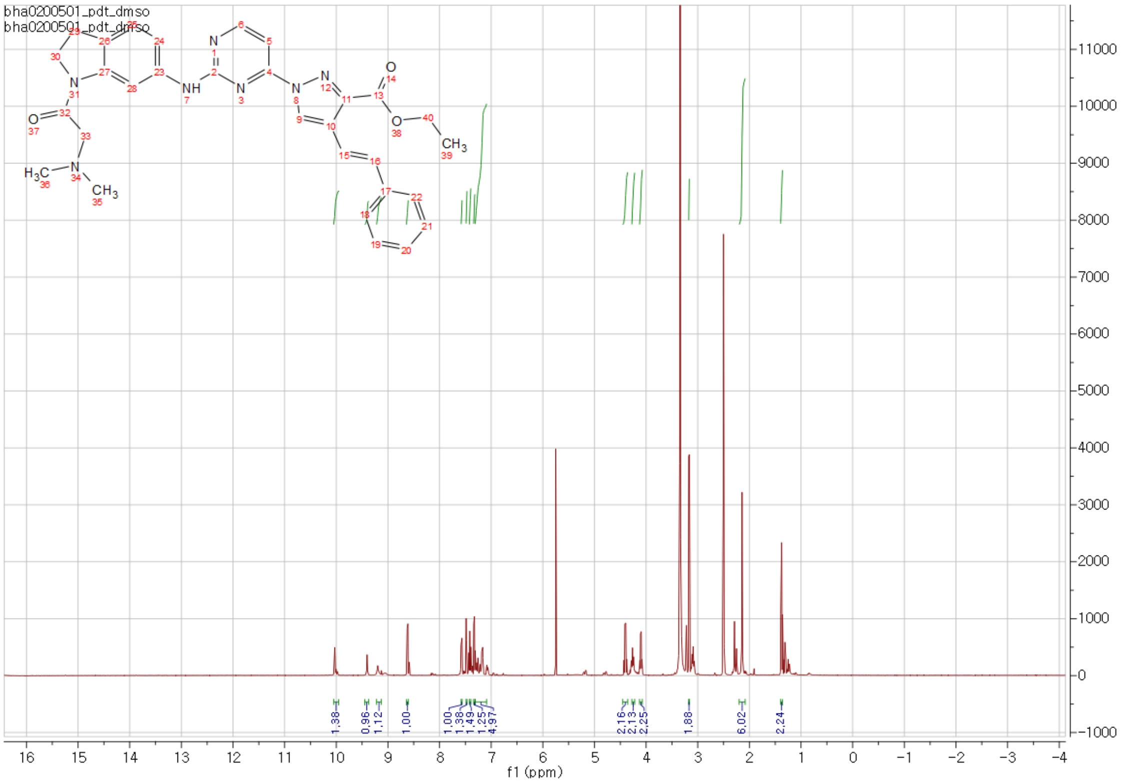

D40

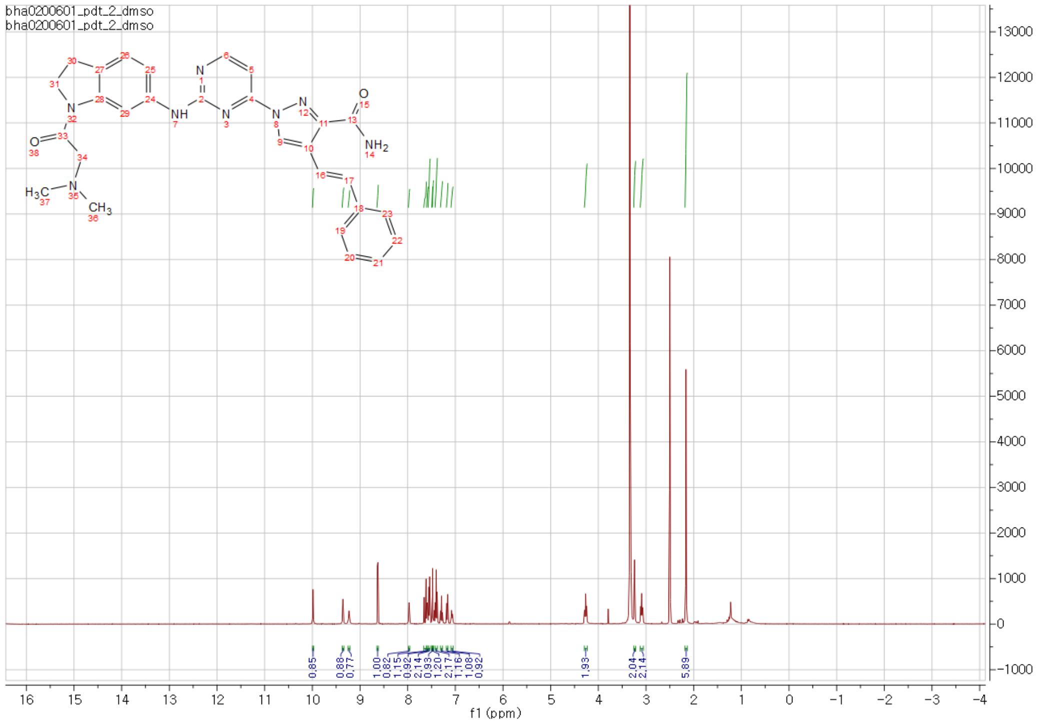

^{13}C NMR Spectra

D39

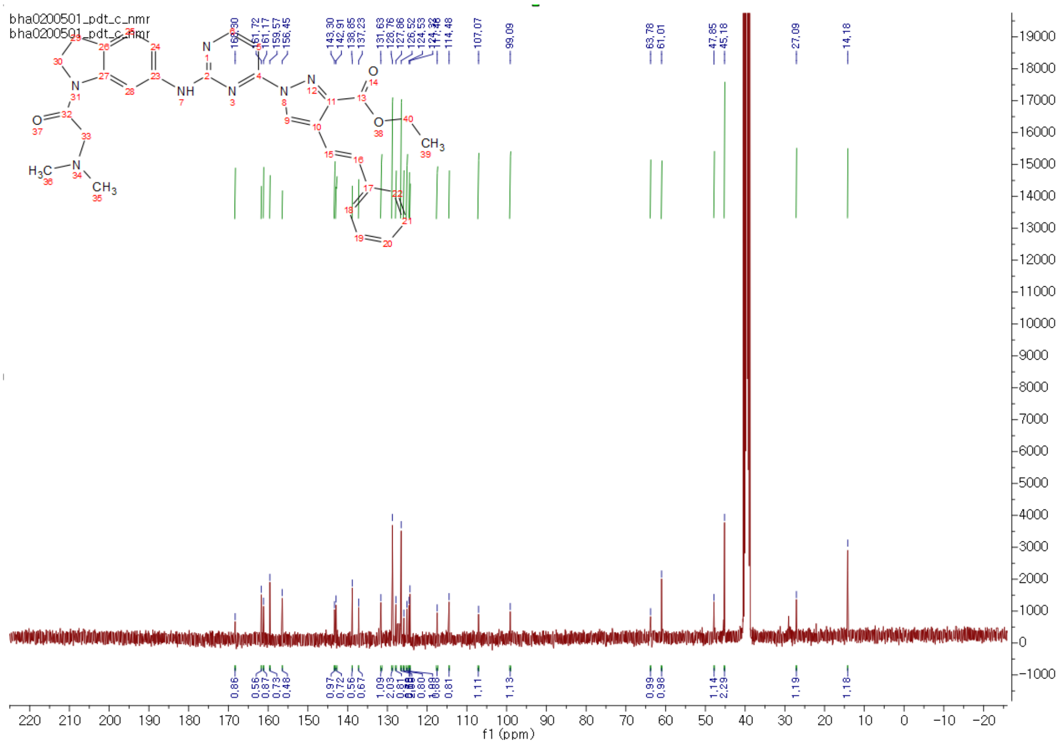

D40

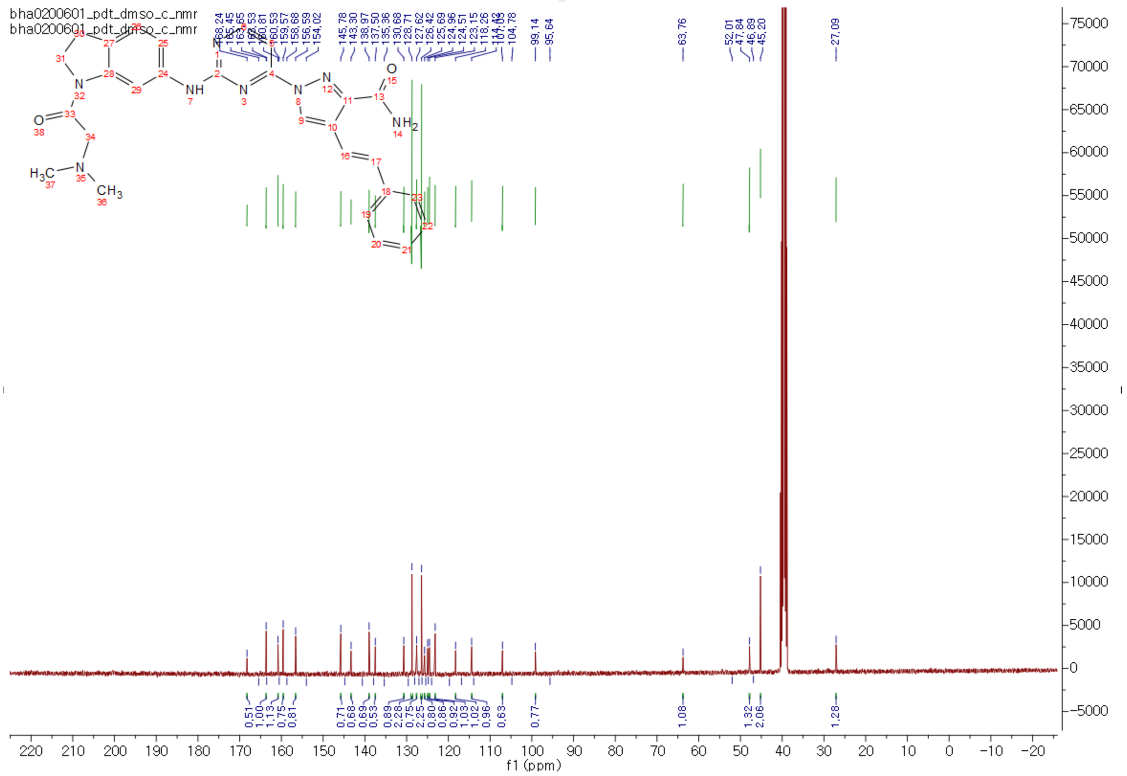

Supplement: Supplementary file 1 [file pharmaceuticals-15-01170-s001.zip › pharmaceuticals-1895097-supplementary.pdf]
